# Environmental preferences of adolescents within a low ecological footprint country

**DOI:** 10.3389/fpsyg.2022.894382

**Published:** 2022-09-26

**Authors:** Franz X. Bogner, Bosque Rafael Suarez

**Affiliations:** ^1^Department of Biology Education, Centre of Math and Science Education (Z-MNU), University of Bayreuth, Bayreuth, Germany; ^2^El Centro de Estudios de Educación Ambiental (CEEA-GEA), Universidad de Ciencias Pedagógicas de La Habana, Havana, Cuba

**Keywords:** attitudes, values, 2-MEV, connectedness with nature, secondary school students, Rasch analysis, principal component analysis (PCA)

## Abstract

As Cuba achieves one of the lowest per capita ecological footprints in the world, the country’s overshoot day was on 1 December 2019, while some European countries already reach this limit in February (e.g., Luxembourg), monitoring the environmental preferences of the Cuban younger generation may offer valuable behavioral or pedagogical insights into such a society. As accepted standardized measures exist in the scales of 2-Major Environmental Values (2-MEV) and the General Ecological Behavior (GEB), both measures are following the necessary psychometric requirement, as they have the unique advantage of repeated independent confirmation (and thus provide an external validity). These captured 40 items of reported behavior originating in six subscales that total in a single main cover score. The first one (2-MEV) monitors individual biocentric and anthropocentric preferences with a 20 item-set by relying upon these two higher-order factors of “Preservation” (PRE) and “Utilization” (UTL). Although many language versions already exist (33 in the case of the 2-MEV) for verifying validities and reliabilities of both scales, a country such as Cuba may affirm that this is due to expected cultural differences as well as their exceptionally low global footprint. Additionally, neither the individual connectedness with nature nor the diurnal preferences within the linear structural model showed a substantial relationship to PRE or UTL. Nevertheless, all the regression scores follow the expected positive or negative directions, albeit not all the fit scores turned out as satisfactorily. Apparently, the applied measures secure a good basis for measuring the attitudinal and behavioral framework, but will need further fine tuning to completely monitor the environmental preferences of Cuban adolescents.

## Introduction

Cuba is a country with one of the least ecological footprints per capita in the world (Global Footprint, 2020): The country’s overshoot day was on December 1, 2019, while European countries such as Germany have already reached this limit by May 3, March 5 in Belgium, or as early as February 16 in Luxembourg. Very few countries rate better than Cuba, such as Ecuador on December 12, 2019 (Global Footprint, 2020). Positioning humanity’s resource consumption into a calendar year illustrates best how the Earth’s resource capacity is overused; the allocated day points to the date when a country’s population is entering its environmental deficit spending (Catton, 1980). In consequence, the Cuban population is living at an almost sustainable level (although with a 1/12 over-usage), while Germany demands resources almost equivalent to three piles of the earth (i.e., 2.96!) (Global Footprint, 2020). Nevertheless, countries such as Germany during the last decades have almost halved their ecological footprints and thus show the effects of decision makers toward using up ecological resources of a country. Although for Cuba we do not have such historical data (and thus the progress history of limiting this threat), the current screenshot pattern is of help for educators to plan their interventions (e.g., van de Wetering et al., 2022) as applying the ecological footprint model illustrates the huge gap between human demand and regeneration potential (Blomqvist et al., 2013). Therefore, we, in different countries, are eating up the Earth’s future resources unequally in operating our current economies. Viewing the range of ecological footprints, Cuba seems to present a promising case, as the societal development of Cuba followed a different path compared to neighboring societies; although its annual GDP increased by 8.82 billion USD and wealth ranks 62nd out of 186 countries (retrieved 2020),^1^ its global footprint, ranks internationally as almost the best (Global Footprint, 2020). In consequence, the chance of the actuality of it achieving ecological sustainability is apparent.

To optimally intervene within this societal conflict, the understanding of a learning program’s effects on adolescent preferences is at least very helpful. In our case we did not yet focus on intervening with educational programs, nevertheless, the present study is planned to act as the basis for that. After decades of discussion about mechanisms to best monitor them (e.g., Kraus, 1995; Leeming et al., 1995), a bi-dimensional model was presented in allowing an individual a preservational and a utilitarian position within a higher order construct based on attitude-sets (Bogner and Wiseman, 1999). Such measures of attitude, toward nature and environment in the 2-Major Environmental Values (2-MEV model; Bogner and Wiseman, 1999, 2002a,^2006^), are overarched by UTL and PRE dimensions. Various European bi-national application studies with different cultural backgrounds (e.g., Bogner and Wiseman, 1998, 2002b; Bogner et al., 2015) assured its validity in limiting the item number to finally just 20. For this purpose, internal cross-validations studies contributed additional strength: (i) A first study brought risk-taking behavior into play (Bogner et al., 2000) in strongly supporting the two orthogonal dimensions: Controlled and cautious gamblers turned out to be high scorers on PRE, while Utilizers failed to control risk-taking behavior. (ii) A second study aligned the personality variables “Psychoticism,” “Extraversion,” and “Neuroticism” together with social desirability, again supporting an orthogonal, two-dimensional representation of both ecological values (Bogner and Wiseman, 2006): Utilizer preferred immediate self-orientated gratification, Preserver a delayed, otherwise-oriented gratification. (iii) A third study introduced Authoritarianism showing a negative correlation of Authoritarianism with PRE and a positive one with UTL (Wiseman et al., 2012).

Besides the described cross-validation studies, the ultimate strength of the 2-MEV model came from repeated independent confirmation studies: (i) Milfont and Duckitt (2004) from a mere psychometric point of view reassured the secondary higher-order structure of PRE and UTL from a purely psychometric point of view. (ii) Johnson and Manoli (2008) had this verified from an educational point of view when searching for an appropriate instrument for evaluating United States-wide earth education programs. (iii) Boeve-de Pauw and van Petegem (2011) assured the 2-MEV structure did this from a pedagogical point of view, while sufficiently assuring the two-dimensional structure as well. (iv) Borchers et al. (2013) coming from a psychological-pedagogical background for third-world sample size, also confirmed the two-factor second-order structure in a study conducted in West Africa. Finally, (v) Braun et al. (2017) reassured the scale’s validity for Asian samples. Furthermore, Bogner (2018) recently aligned assured the scale’s validity even when emotional appreciation of nature to the 2-MEV without losing the structure’s validity (Gooch, 1995; Casey and Scott, 2006; Kibbe et al., 2014; Sarner and Long, 2018; Torkar and Bogner, 2019). Currently, to our knowledge, the scale exists in 33 language versions all over the world.

Monitoring individual behavior has also seen many attempts prompting fierce discussions within psychology research for some time. Its assessment challenged psychology’s conventional wisdom as verbal claims are collected instead of actual behavioral observations (e.g., Ajzen, 1991; Stern, 2000; Vining and Ebreo, 2002). Kaiser et al. (2010), for instance, developed a measure containing a composite of various reported conservation behaviors. By employing this General Ecological Behavior (GEB) scale in a traditional planned behavior framework, its construct validity was corroborated with a virtually perfect intention-behavior link (Kaiser et al., 2007). Ecological behavior within this context is understood as action contributing toward environmental PRE by including behaviors such as recycling and composting, energy and water conservation, political activism, consumerism, commitment to environmental organizations, and so forth. The so-called GEB scale is favored as not being bound to a particular set of ecological behaviors or to a particular questionnaire response format (Lange and Dewitte, 2019). Although originally developed on the basis of six main behavioral preferences the GEB scale has been shown to cover essential facets of human behavior (Kaiser and Wilson, 2004). Subsequently, all sub-behaviors have been analyzed with respect to their relationships revealing a tremendous overlap of correlations. Kaiser and Wilson (2004) therefore suggested a merging of all subscale scores to one portraying the “GEB.” Similarly important, an age-appropriate modification made the GEB scale suitable for adolescents (Kaiser et al., 2007). Although many approaches are available in the literature (e.g., Sparksa et al., 2022), for the educational application we increasingly had built upon the GEB due to its specific age adjustment (Kaiser et al., 2007).

Connectedness with Nature is a simple measure, which repeatedly has shown its validity as the Inclusion of Nature in Self scale (INS) (Mayer and Frantz, 2004; Schultz et al., 2004; Liefländer et al., 2015). This is also true for its cultural context validity (e.g., Navarro et al., 2017). Some studies provided a convincing explanation about times in life at which a person is most susceptible to consolidating a strong connectedness to nature (e.g., Wells and Lekies, 2006; Ernst and Theimer, 2011): Especially young children, in general, have been shown highly connected with nature, just as high as environmental activists. Connectedness to nature is regarded to portray the interpersonal relationship between an individual and another person characterized by an overlapping of the cognitive representation of the self and another person. Visualizing this relationship repeatedly had shown its usability in educational contexts (Randler and Bogner, 2009; Kossack and Bogner, 2012; Sellmann and Bogner, 2013).

Circadian preferences are well-known to influence behaviors for given times of the day supporting an individual’s physical peak performances (Randler et al., 2017): Morning persons get up and go to bed early, while evening persons get up and go to bed late, thus, morning persons reach their peak performance during the morning, while evening people reach it during the afternoon or even during the night. A recent study with Irish school children monitored the relationships between the environmental values and MESC scale reporting a small gender difference and again some small positive correlations (Raab et al., 2018): Not surprisingly, “early birds” were shown to have better links to protective and appreciative attitudes toward nature.

The objectives of our current study were (i) to apply a combined attitudinal and behavioral questionnaire to a Cuban secondary school population and subsequently analyze the relationships of both constructs. (ii) To monitor variables such as connectedness (inclusion) with nature and the circadian preferences and relates them with (i) above, (iii) to conclude the field for appropriately preparing subsequent biodiversity implementation studies according to the Cuban adolescents.

## Materials and procedures

Our sample consisted of 348 secondary school students (57.4% men, aged 14.9 years) of the urban Havana region. All participants completed the paper-and-pencil questionnaire once during school hours; the individual response sheets were digitalized *via* scanning and, if needed, by hand. The 2-MEV measure followed a five-point response pattern with “Strongly disagree, Disagree, Not sure/neutral, Agree, and Strongly Agree,” and the GEB measure followed a frequency response pattern of “Never, Seldom, Occasionally, Often, and Always (GEB). Both scales previously had shown their suitability to different languages and cultures within studies by different authors. The GEB is an instrument to assess self-reported behavior-based attitudes through Rasch modeling (Kaiser and Wilson, 2004). According to the Campbell paradigm, item difficulty and individual disposition affect attitudes and behavior (Kaiser et al., 2010), we applied the probabilistic Rasch measurement acknowledging individual engagement (number of items answered correctly) and item difficulty (number of people answering the item correctly) and eventually allocating each participant a logit (Bond and Fox, 2010). To conduct the Rasch analysis, we used the software ConQuest. The INS scale consisted of two circles intertwining with each other according to the individual’s estimate of its closeness with nature, the diurnal preferences were monitored with the scale of Randler et al. (2017).

Although our dataset was not normally distributed, due to the sample size we applied a parametric test as the Statistical Program for Social Sciences (SPSS, 24 version) yielded identical results for all (non-) parametric calculations. We applied IBM SPSS AMOS 24 (Analysis of Moment Structures; Arbuckle, 1997) to calculate confirmatory factor analyses, to relate the attitudinal scales through path modeling in a multivariate context. All AMOS figures display standardized values and are based on the Maximum Likelihood Solution. We report absolute and incremental fit indices to estimate the adequacy of our postulated model. Hooper et al. (2008) recommended reporting the Chi-Square, the degrees of freedom, the *p*-value, the Root-mean-square Error of Approximation (RMSEA) as an incremental index, and the Comparative Fit Index (CFI) as an absolute index. They asked for a low Chi-Square (χ^2^) in relation to the degrees of freedom with a non-significant *p*-value (*p* > 0.05). To take the sample size into consideration, we report the relative χ^2^ (CMIN/DF), which should be lower than 5. The CFI is recommended >0.9, and the RMSEA <0.07 (see Wheaton et al., 1977).

## Results

To examine the first-order factors, the 2-MEV responses were subjected to a principal component analysis followed by oblique rotation (see examples Table 1) and were again subjected to principal factor analysis (PCA), revealing the expected structure of a PRE and UTL factor including the Appreciation subscales (as proposed in Bogner, 2018) (not shown here). The model was fitted using a simultaneous maximum likelihood second-order factor analysis *via* structural equation methods (see Arbuckle, 1997). The Kaiser–Meyer–Olkin measure of sampling adequacy (0.826) was high and the Bartlett-test of Sphericity was significant (*p* < 0.001) (Field, 2018). Hence we accepted the bi-factorial model of Bogner and Wiseman (1999, 2002a).

**TABLE 1:** Listing of the five best item examples of Preservation, Utilization, and Appreciation 2-Major Environmental Values (2-MEV) as well as reported ecological behavior General Ecological Behavior (GEB) with different difficulties.

**Preservation**
It is interesting to know what kinds of creatures live in the lagoons or in the rivers.
Humanity will disappear if we do not live in harmony with nature.
Human beings do not have the right to change nature as they see fit.
Not only economically important plants and animals need protection.
**Utilization**
We need to clear forests in order to grow crops.
Nature is always able to restore itself.
Our planet has unlimited resources.
People worry too much about pollution.
**Appreciation**
I take time to consciously smell flowers.
I enjoy gardening.
Listening to the sounds of nature makes me relax.
I personally take care of plants.
**General Ecological Behavior**
As the last person to leave a room, I switch off the lights.
I ride a bicycle, take public transportation or walk to school.
If I am offered a plastic bag in a store, I take it.
For making notes, I take paper that is already used on one side.

**FIGURE 1:**
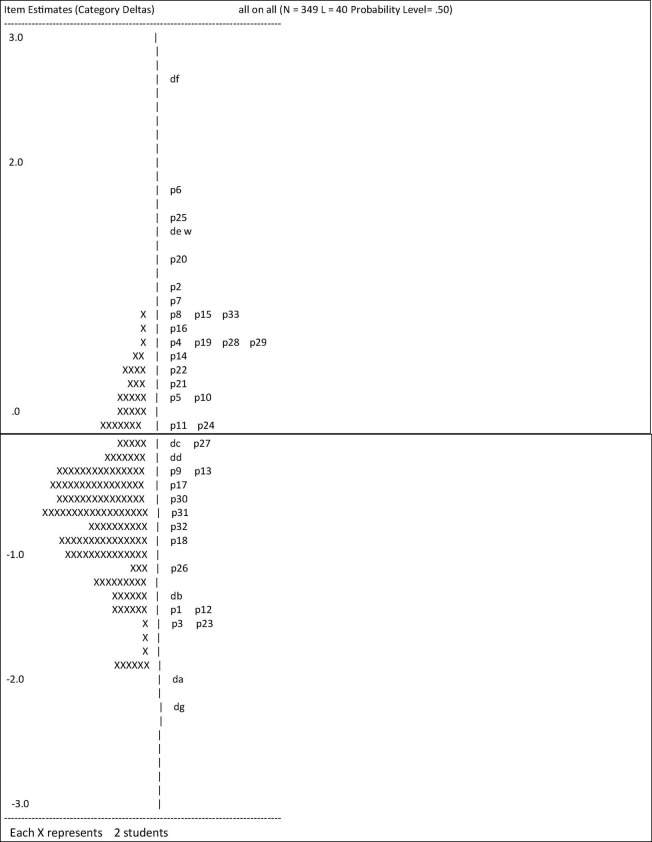
Rasch analyzed the distribution of the 40 General Ecological Behavior (GEB)-items pointing to a smooth fit- statistics (each X represents 2 students).

In line with the original design of the instrument, we analyzed the data with the Rasch model (Figure 1). The fit of the 40 items scored with a mean MS of 1.14 (SD = 0.19). They lie within the cut-off levels of Bond and Fox (2010), who suggest 0.6 at the lower and 1.3 at the upper end. The fit statistics of persons are accepted as just 7.1% (*n* = 24) are not describable with the Rasch model. However, the reliability score of 0.53 is rather low in pointing to some failure to discriminate within the sample.

**FIGURE 2:**
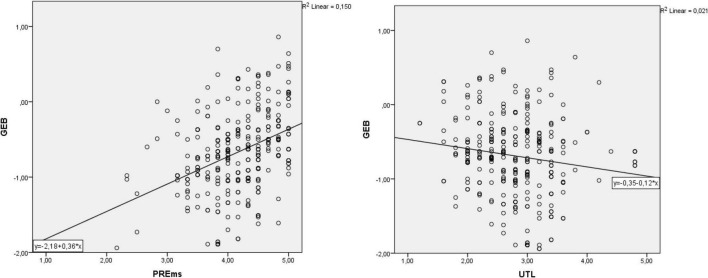
Correlation pattern of preservation vs behaviour and utilisation vs behaviour.

Given the two factor scores of PRE and UTL as well as the Rasch-scored Behavior score we plotted both correlation matrices unveiling the expected pattern: As the higher an individual scores in PRE the more likely he/she is to score higher in the behavior, versus the other way round for UTL, however, with a much lower correlation (Figure 2).

**FIGURE 3:**
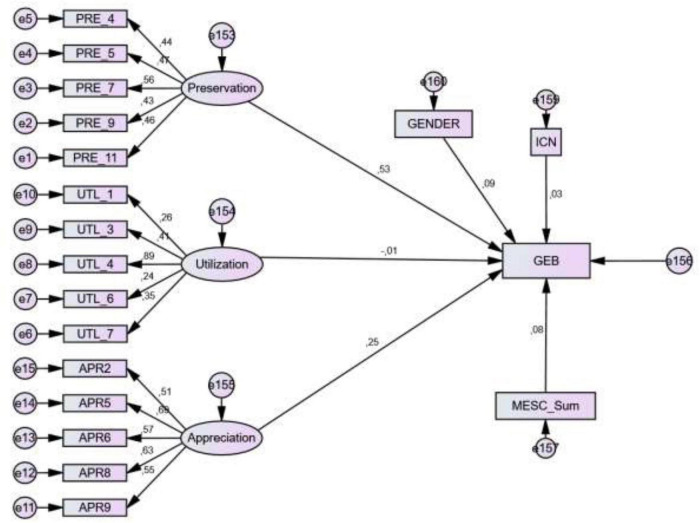
A path analysis of variables involved in the reported behavior General Ecological Behavior (GEB).

The subsequent path model (Figure 3) displays the Rasch analyzed GEB score in relation to the factor analyzed environmental value scores, the INS, the morningness–eveningness preference as well as gender. PRE and Appreciation provide the highest forecast scores, while almost all other variables contribute negligibly. Nevertheless, the model’s fit-data score is acceptable: Chi-square = 496.334; df = 169; *p* < 0.001, CMIN/DF = 2.923, CFI = 0.666, RMSEA = 0.074.

## Discussion

The Cuban sample finding in principle is in line with earlier studies of a quite different country’s attitude-sets displayed often by results from other independent research groups (see details Introduction). Both scales have undergone a consequent development laying a solid theoretical foundation (e.g., Bogner and Wiseman, 2002a; Kaiser and Wilson, 2004; Bogner and Wiseman, 2006). For the 2-MEV, several binational applications assured a cultural stability (e.g., Bogner and Wiseman, 1998, 2002b) or a suitable rural/urban coverage (Bogner and Wiseman, 1997). Two studies have previously specifically applied the 2-MEV scale to third-world samples, Boeve-de Pauw and van Petegem (2011) in Guatemala and Vietnam as well as Borchers et al. (2013) in the Ivory Coast. Up to now, knowledge has come from 33 different language versions although many more may exist without publication (Bogner, 2018). In consequence, both scales overcome the frequent fundamental problem of empirical research contexts where agreed test instruments are commonly lacking and thus do not allow comparability of research study results. Nevertheless, for its valid application, each item must show that it is loading on the theoretically derived factor and is designed in the same way. Establishing just reliability or construct validity is often not justified as the assumption needs explicit proof by testing for factorial invariance (Milfont and Duckitt, 2004). Factorial invariance would point to a lack of equivalence of factors in both contexts: measurement invariance and structural invariance (Byrne et al., 1989). As measurement invariance assesses invariance tendencies of the basic model structure, factor loadings, and error variances, structural invariance does this with the invariance of factor variances, factor co-variances, and factor means.

The interrelationship of both scales has repeatedly shown its potential validity to grasp the adolescents’ preferences as well as its capacity to monitor and frame educational initiatives. First, it is important to highlight the stability of measures within various contexts (e.g., Johnson and Manoli, 2010; Schumm and Bogner, 2016; Kleespies and Dierkes, 2020; Maurer et al., 2020). This was even true for diverse cross-cultural contexts, for instance, as recently applied in China (Liu and Chen, 2019). Any educational initiative whether environmental education (EE) or education for sustainable development (ESD) aims to support the preferences of young people in order to oust our current anthropocentric way of life in favor of more eco-centric lifestyles. For generations, all human beings have been extracting wealth from nature with the intention of achieving continual growth:- with this as the underlying philosophy of all stakeholders. Moreover, the resulting destruction of natural habitats aligned with increasing worldwide pollution and environmental over-exploitation by human activities alongside increasing population densities has become obvious (Foley et al., 2005). Acquisition of natural resources for immediate human needs occurs at the expense of degrading natural and environmental conditions. As exponentially growing world populations are aligned with increasingly intensive land usage it places extraordinary pressure on ecosystems so alarming naturalists to seek a counteraction. Through many other channels, the awareness of young people must shoulder responsibility for slowing down or even stopping this exploitation (Bogner, 1999). Many grass-roots pioneers have laid (philosophical) foundations going back to Muir (1916) or Dewey (1929) or to Leopold (1949) or later on to Carson (1962) in order just to name some of the major forerunners.

Preservation and Appreciation positively affect an individual’s behavior which is quite in line with recent path analysis studies (Baierl et al., 2021b): The higher that subscale level is the more likely students are assigned to adopt more positive behavior. Attitudes level out performative costs (Bond and Fox, 2010) and assign themself to pro-environmental behavioral tendencies (Roczen et al., 2014; Allen et al., 2018). Compared to the strong pro-environmental preference the other monitored variables such as UTL, Inclusion with nature or morningness–eveningness-preferences provide negligible influence which is, in contrast, the recent studies (e.g., Jacobs and McConnel, 2022).

Even gender does not substantially explain the behavioral target variable. As the situation is balanced, some studies show an influence that others do not, and this relationship may fit into the literature body (e.g., Johnson and Manoli, 2008). This is even more likely as most recent studies came to the latter results which is lacking a gender influence. For instance, Baierl et al. (2021a) showed for a United States secondary school sample stronger relationships of fascination, motivation, or knowledge than gender or parental education contribute to it. Other studies such as Lau and Roeser (2002) similarly showed such a pattern. In other contexts, such as technology or science education, gender should also be considered as an important predictor which has made gender issues become frequent targets of research (Acker and Oatley, 1993; Evans et al., 2007; Mayer-Smith et al., 2000).

A major question in almost all related environmental education studies is the overall desire to redirect adolescent behavior in a more ecological direction (e.g., Hines et al., 1987; Leeming et al., 1995; Bogner, 1999; Schneller et al., 2015; Bissinger and Bogner, 2018) as educational approaches generally focus on its improvement (e.g. Liefländer and Bogner, 2014, 2018; Schneller et al., 2015; Bissinger and Bogner, 2018). Especially outdoor educational attempts presumably provide appropriate triggers to coping with that environmental challenge we doubtless face nowadays in the need to replace traditional exploiting approaches. Education in general, and especially environmental education, must build upon existing preferences, especially in a country such as Cuba (or Ecuador) with its marvelous low carbon footprints may offer new channels to monitor and understand promising channels to overcome this well-known dilemma. Pupils coming from a background less exploitative of nature are apparently more likely to offer sensitivity to appropriate educational access (e.g., Bogner and Wiseman, 1997). As the current environmental dilemma must be decoded as a conflict of traditions passed on to the young by cultural mechanisms, the overall background of extracting and exploiting for our own advantages may offer more likely chances for a society to achieve change. Follow-up studies with Cuban samples therefore may provide new insights into what educational programs will be more effective in achieving the desired shifts in paradigms. Within the view that Romance language cultures countries may see nature as mastered, without distinguishing between what is man’s fact and what is the fact of nature (Ducamre and Couvet, 2020), surprises are not unlikely. As a first steppingstone of information the further research line is twofold: First, to broaden the variable groundwork which, second, may allow researcher/educator to build upon a variability pool according to their specific educational foci.

### Limitations

Although the study was conducted with great care, some limitations may apply. One methodological limitation is the survey group which was only from one representative sample, the selection of this group being concentrated on the urban capital region. Additionally, our study does not reflect the full age structure as the sampling was undertaken class-wise and school-wise. Another methodological limitation might arise from the use of shortened scales although in all subscales a clear structure appeared. Since the questionnaire was answered during regular courses, it was necessary to keep it as short as possible. Although the reliability, validity, and correct measurement were proven, it is possible that some information was lost due to that reduction. Additionally, the CFI value is very low given the limit of 0.9 in the methods section while the RMSEA value scores slightly above the mentioned limit. We regard both results as a limitation (internally explaining this with the small sample) but consider this information useful for upcoming further studies in Cuba.

## Data availability statement

The raw data supporting the conclusions of this article will be made available by the authors, without undue reservation.

## Ethics statement

The studies involving human participants were reviewed and approved by the Universidad de Ciencias Pedagógicas Enrique José Varona, de La Habana, Cuba. Written informed consent to participate in this study was provided by the participants’ legal guardian/next of kin.

## Author contributions

BS initiated and supervised the data collection. FB completed the statistical analysis and initiated the first manuscript draft. Both authors cooperatively designed the study and contributed to the final version of the manuscript.

## Footnotes

1
http://www.data.worldbank.org
